# Optimization of high molecular weight DNA extraction methods in shrimp for a long-read sequencing platform

**DOI:** 10.7717/peerj.10340

**Published:** 2020-11-13

**Authors:** Pacharaporn Angthong, Tanaporn Uengwetwanit, Wirulda Pootakham, Kanchana Sittikankaew, Chutima Sonthirod, Duangjai Sangsrakru, Thippawan Yoocha, Intawat Nookaew, Thidathip Wongsurawat, Piroon Jenjaroenpun, Wanilada Rungrassamee, Nitsara Karoonuthaisiri

**Affiliations:** 1 Microarray Research Team, National Center for Genetic Engineering and Biotechnology (BIOTEC), National Science and Technology Development Agency, Pathum Thani, Thailand; 2 National Omics Center, National Science and Technology Development Agency, Pathum Thani, Thailand; 3 Department of Biomedical Informatics, College of Medicine, University of Arkansas for Medical Sciences, Little Rock, AR, United States of America; 4 Division of Bioinformatics and Data Management for Research, Department of Research and Development, Faculty of Medicine, Siriraj Hospital, Mahidol University, Bangkok, Thailand

**Keywords:** DNA extraction method, Long-read sequencing platform, Marine organism

## Abstract

Marine organisms are important to global food security as they are the largest source of animal proteins feeding mankind. Genomics-assisted aquaculture can increase yield while preserving the environment to ensure sufficient and sustainable production for global food security. However, only few high-quality genome sequences of marine organisms, especially shellfish, are available to the public partly because of the difficulty in the sequence assembly due to the complex nature of their genomes. A key step for a successful genome sequencing is the preparation of high-quality high molecular weight (HMW) genomic DNA. This study evaluated the effectiveness of five DNA extraction protocols (CTAB, Genomic-tip, Mollusc DNA, TIANamp Marine Animals DNA, and Sbeadex livestock kits) in obtaining shrimp HMW DNA for a long-read sequencing platform. DNA samples were assessed for quality and quantity using a Qubit fluorometer, NanoDrop spectrophotometer and pulsed-field gel electrophoresis. Among the five extraction methods examined without further optimization, the Genomic-tip kit yielded genomic DNA with the highest quality. However, further modifications of these established protocols might yield even better DNA quality and quantity. To further investigate whether the obtained genomic DNA could be used in a long-read sequencing application, DNA samples from the top three extraction methods (CTAB method, Genomic-tip and Mollusc DNA kits) were used for Pacific Biosciences (PacBio) library construction and sequencing. Genomic DNA obtained from Genomic-tip and Mollusc DNA kits allowed successful library construction, while the DNA obtained from the CTAB method did not. Genomic DNA isolated using the Genomic-tip kit yielded a higher number of long reads (N50 of 14.57 Kb) than those obtained from Mollusc DNA kits (N50 of 9.74 Kb). Thus, this study identified an effective extraction method for high-quality HMW genomic DNA of shrimp that can be applied to other marine organisms for a long-read sequencing platform.

## Introduction

Seafood is the largest source of animal protein (FAO, 2016), making aquaculture the world’s fastest growing food-production (Anderson et al., 2017). The industry strives to increase production capacity while reducing cost by utilizing modern technologies. Genome sequence can serve as an important tool for the identification of key pathways to guide potential functions (Collins et al., 2003; Ramanan et al., 2012). Genome sequence has enabled the identification of single nucleotide polymorphisms (SNPs) and genetic markers for desirable traits (Zwane et al., 2019) such as productivity, disease resistance and reproductive maturation (Guppy et al., 2018). For instance, specific SNPs for growth performance and immunity traits have been identified and utilized for breeding programs assisted by genomic selection in Blanco Orejinegro cattle (Martínez et al., 2010; Martínez et al., 2016), chicken (Li et al., 2013; Xie et al., 2012) and pig (Geraci et al., 2019; Yang et al., 2019). Genomics has indeed contributed to the exponential growth of terrestrial animal production, yet it has not been fully exploited in aquaculture industry due to several challenges in obtaining whole genome sequences of marine organisms.

With continuing progress of sequencing technology, whole genome assemblies of few marine organisms are now available such as Atlantic salmon (*Salmo salar*) (Davidson et al., 2010), Atlantic cod (*Gadus morhua*) (Star et al., 2011), Pacific oyster (*Crassostrea gigas*) (Zhang et al., 2012), Pacific white shrimp (*Litopenaeus vannamei*) (Zhang et al., 2019), Black tiger shrimp (*Penaeus monodon*) (Uengwetwanit et al., 2020) and Rainbow trout (*Oncorhynchus mykiss*) (Berthelot et al., 2014). Although whole genome sequencing and de novo assembly have become a routine method for functional genomics studies, genome sequencing of non-model organisms with a large proportion of long repetitive sequences such as marine invertebrates remains challenging. Recently, the long-read sequencing platforms present an alternative approach to overcome those challenges with the following advantages: (i) longer read length to facilitate de novo assembly and enable direct detection of haplotypes and even whole chromosome phasing; (ii) higher consensus accuracy to enable rare variant detection; (iii) low cost per run; and (iv) provide the epigenetics information (Metzker, 2010; Schadt, Turner & Kasarskis, 2010).

With an ability to span repetitive sequences, long-read sequencing technology presents a promising alternative to obtain genome assemblies for marine species (Amarasinghe et al., 2020; Pollard et al., 2018). High-quality genomic DNA is the key to allow the utilization of the long-read sequencing technology. However, obtaining such high-quality genomic DNA from crustaceans faces several challenges because crustacean DNA is easily degraded due to endonuclease enzyme (Rahman, Schmidt & Hughes, 2017) and the purity can be jeopardized by high amounts of polysaccharides and polyphenolic proteins, which can inhibit subsequent molecular applications (Panova et al., 2016).

Despite many DNA extraction methods available, the efficiency of methods depends on specimen and preservation (Abdel-Latif & Osman, 2017; Psifidi et al., 2015). DNA extraction methods in marine organisms have been evaluated for PCR application (Chakraborty, Saha & Ananthram, 2020; Jasrotia & Langer, 2019; Nadia & Sadok, 2017; Pereira et al., 2011) but not for long-read sequencing.

This study therefore aimed to evaluate DNA extraction methods that yielded high quality genomic DNA, enabling full exploitation of the long-read sequencing technology. We employed the black tiger shrimp (*Penaeus monodon*) as a model marine organism as it is one of the most widely cultured shrimp species in the world, accounting for 52% of total global farm production (FAO, 2016).

## Materials & Methods

### Shrimp collection

Four-month-old black tiger shrimps (*n* = 4) were obtained from a local farm in Prachuap Khiri Khan, Thailand. Muscle tissue from each shrimp was collected and immediately frozen in liquid nitrogen and stored at −80 °C prior to DNA extraction.

### Genomic DNA extraction methods

Five DNA extraction methods namely (I) CTAB method, (II) Genomic-tip 100/G kit (Qiagen, Germany), (III) E.Z.N.A.^^®^^ Mollusc DNA kit (Omega bio-tek, USA), (IV) TIANamp Marine Animals DNA kit (Tinagen, China) and (V) Sbeadex livestock kit (LGC, Germany) were evaluated for genomic DNA preparation (4 biological replicates/extraction method).

Frozen shrimp muscle was transferred to mortar containing liquid nitrogen and pulverized. All tissue samples were immediately processed according to the instruction of each method as follows:

#### Cetyltrimethyl ammonium bromide (CTAB) method

CTAB method used in this study followed a previously described protocol (Winnepenninckx, Backeljau & De Wachte, 1993). Dried powder of frozen shrimp tissue (100.02 ± 0.04 mg) was mixed with 800 µL of an extraction buffer (2% (w/v) CTAB, 1% (w/v) polyvinylpyrpolidone, 1.4 M NaCl, 20 mM EDTA, pH 8.0; 100 mM Tris–HCl, pH 8.0) by gently inverting the tube before 20% SDS (20 µL) was added and incubated at 65 °C for 10 min. RNA was removed by incubating with 10 µL of 10 mg/mL RNase A (final concentration of 0.12 mg/mL) at 37 °C for 30 min. Subsequently, 50 µL of *β*-mercaptoethanol and 500 µL of chloroform were added and mixed by inverting. The mixture was centrifuged at 10,000 g for 5 min at 10 °C. The upper aqueous phase was transferred and extracted with 500 µL of chloroform:isoamyl alcohol (24:1) by inverting and centrifugation as before. The chloroform:isoamyl alcohol extraction step was repeated twice before DNA was precipitated with 2 volumes of 100% ethanol. The sample was centrifuged at 12,000 g for 10 min at 10 °C and the supernatant was discarded. The DNA pellet was washed with one mL of 70% ethanol, centrifuged at 12,000 g for 5 min at 10 °C, air-dried and resuspended in 50 µL of TE buffer (10 mM Tris–HCl, pH 8.0; 1 mM EDTA, pH 8.0).

#### Genomic-tip kit

DNA was extracted according to the manufacturer’s protocol. Briefly, 100.04 ± 0.02 mg of shrimp muscle was mixed with 10 mL of buffer G2 containing 20 µL of 100 mg/mL RNase A (final concentration of 0.2 mg/mL) and 500 µL of 20 mg/mL Proteinase K (final concentration of 1 mg/mL) and incubated at 50 °C for 4 h or until the lysate was clear. Qiagen Genomic-tip 100/G column was equilibrated with five mL of buffer QBT and emptied by gravity flow. The lysate sample was then added into the column and allowed the column to empty by gravity flow. The column was washed twice with 7.5 mL of buffer QC before eluting genomic DNA twice with five mL of 50 °C buffer QF. The eluted genomic DNA was precipitated with four mL of isopropanol, centrifuged at 5,000 g for 15 min at 4 °C. The DNA pellet was washed with five mL of cold 70% ethanol by centrifugation at 5,000 g for 10 min at 4 °C. The DNA pellet was air-dried for 10 min and then resuspended with 100 µL of TE buffer.

#### E.Z.N.A^^®^^ Mollusc DNA kit

DNA was extracted following the manufacturer’s protocol. Briefly, 50.03 ± 0.03 mg of muscle tissue was mixed with 350 µL of ML buffer. Proteinase K solution (25 µL of 20 mg/mL, final concentration of 1.4 mg/mL) was added to the mixture to digest protein contamination by incubating at 37 °C for 16 h before mixing with one volume of chloroform:isoamyl alcohol (24:1). The reaction was centrifuged at 15,000 g for 10 min at 25 °C. The upper layer of the supernatant was transferred to one volume of BL buffer before 10 µL of 10 mg/mL RNase A were added, incubated at 70 °C for 10 min and cooled-down to room temperature. One volume of 100% ethanol was added and mixed. The HiBind DNA Mini Column was prepared by adding 100 µL of 3M NaOH and centrifuged at 15,000 g for 1 min at 25 °C before 700 µL of sample was transferred to the column. The column was centrifuged and the filtrate was discarded. These steps were repeated until the remaining samples were applied to the column. The column was transferred into a collection tube before adding HBC Buffer (500 µL). The mixture was centrifuged at 13,000 g for 1 min at 25 °C and the filtrate was discarded. The column was washed twice with DNA Wash Buffer (700 µL), followed by centrifugation at 13,000 g for 2 min at 25 °C to dry the membrane. DNA was eluted with the Elution Buffer (50 µL) that had been heated to 70 °C, incubated at room temperature for 5 min and centrifuged at 13,000 g for 2 min at 25 °C.

#### TIANamp Marine Animals DNA kit

DNA was extracted following the manufacturer’s protocol. Briefly, 30.02 ± 0.03 mg of muscle tissue was mixed with 200 µL of Buffer GA by vortexing. RNA was removed by adding 4 µL of 100 mg/mL RNase A (final concentration of 2 mg/mL) at room temperature for 5 min. Proteinase K (20 µL of 20 mg/mL, final concentration of 2 mg/mL) was added and incubated at 56 °C for 3 h. The mixture was added with 200 µL of Buffer GB and incubated at 70 °C for 10 min before 200 µL of 100% ethanol was added. The mixture was transferred into Spin Column CB3 and centrifuged at 13,000 g for 30 s. The column was washed twice with 500 µL of Buffer GD and one time with 600 µL of Buffer PW by centrifugation at 13,000 g for 30 s. The empty column was centrifuged at 13,000 g for 2 min to dry the membrane before DNA was eluted by 50 µL of Buffer TE and centrifuged at 13,000 g for 2 min.

#### Sbeadex livestock kit

DNA was extracted following the manufacturer’s protocol. Briefly, 50.03 ± 0.03 mg of muscle was mixed with 120 µL of PVP buffer, 10 µL of 20 mg/mL protease solution (final concentration of 1.6 mg/mL) and 4.8 µL of debris capture beads and incubated at 55 °C for 2 h. One volume of lysis buffer SB and 5 µL of 20 mg/mL protease solution (final concentration of 0.8 mg/mL) were added to the mixture and incubated at 55 °C for 1 h before centrifugation at 13,000 g at 25 °C for 10 min. The upper aqueous phase was transferred to 180 µL of binding mix and incubated at room temperature for 5 min with constant shaking. The tube was placed in magnetic rack until solution appeared clear, and the supernatant was removed. The beads were washed with 400 µL of wash buffer BN1, wash buffer TN1 and wash buffer TN2, respectively by gently vortexing for 10 min. The tube was placed in a magnetic rack until solution became clear, and the supernatant was removed. The DNA was eluted by 50 µL of Elution buffer AMP and incubated at 55 °C for 10 min. The tube was placed in a magnetic rack until solution appeared clear, and DNA solution was collected to a new tube.

### DNA purification

Each genomic DNA sample was cleaned up using AMPure PB bead (Pacific Biosciences, USA) to remove residual genomic DNA isolation reagents prior to the library preparation by adding 0.45 volume of AMPure PB bead to DNA sample. The mixture was incubated by gently vortexing for 10 min, and tubes were placed in magnetic rack until solution appeared clear. The supernatant was removed and washed AMPure PB bead twice with one mL of freshly prepared 70% ethanol. DNA samples were eluted by adding elution buffer and incubated by gently vortexing for 5 min. Tubes were placed in the magnetic rack until solution became clear, and DNA solutions were collected to a new tube.

### Assessment of DNA quantity and quality

The extracted DNA by each DNA extraction method was quantified by NanoDrop 8000 spectrophotometer V2.3.2 (Thermo Fisher Science, USA) and Qubit dsDNA BR Assay kit (Invitrogen, USA) using Qubit 2.0 Fluorometer (Invitrogen, USA). The DNA yields of shrimp muscle from different extraction methods were statistically tested using one-way analysis of variance (ANOVA) followed by Duncan’s new multiple range test in IBM SPSS statistics 23.0. The DNA quality and integrity were visualized by UV light after electrophoresis in 0.75% of SeaKem^^®^^ Gold Agarose (Lonza, USA) for pulsed-field gel electrophoresis at 80 volts for 9 h in 0.5x KBB buffer (51 mM Tris, 28 mM TASP, 0.08 mM EDTA, pH 8.7) (Sage Science, USA) containing SYBR Safe DNA gel staining (Invitrogen, USA).

### Genomic sequencing analysis

PacBio sequencing was performed at NovogeneAIT (Singapore) following the PacBio’s protocol (Anthony & Kin, 2015; Pacific Biosciences of California, 2014). High-quality HMW DNA obtained was used to generate a 20-kb SMRTbell library. The SMRTbell library itself was produced by ligating universal hairpin adapters onto double-stranded DNA fragments. The hairpin dimers formed during this process were removed at the end of the protocol using a magnetic bead (AMPure PB) purification step. The final step of the protocol was to remove failed ligation products through the use of exonucleases. After the exonuclease and AMPure PB purification steps, sequencing primer was annealed to the SMRTbell templates following by the binding of polymerase to the annealed templates. According to the effective concentration of library and output requirements, the sample was sequenced on PacBio Sequel platform. The original sequencing reads (called polymerase reads) were processed by the software SMRTlink to filter reads with minimum read quality score of 0.8 and to generate subreads. Each polymerase read was partitioned to form one or more subreads, which contained sequences from a single pass of polymerase on a single strand of an insert within a SMRTbell™ template and no adapter sequences. The subreads were used for further analysis.

### Data Availability

The genomic data is available in the NCBI under the accession number: PRJNA611030.

## Results

### DNA quantity

**Figure 1:**
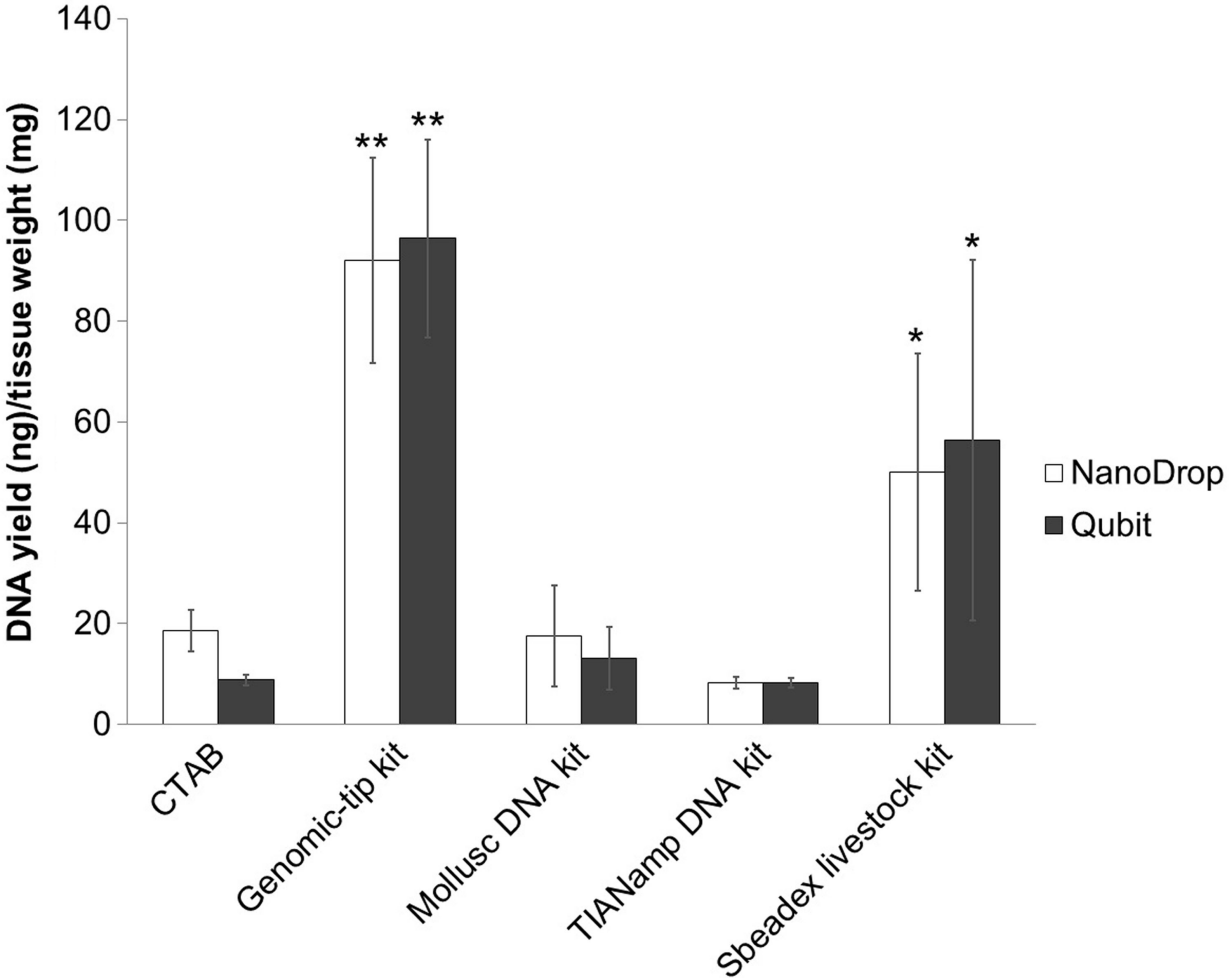
Average DNA yield measured with NanoDrop spectrophotometer and Qubit Fluorometer of shrimp muscle using different DNA extraction methods after purified by AMPure PB bead. Error bars indicate standard deviation of the mean from four replicates. The asterisk indicates significantly different level among the extraction methods: * *P* < 0.05 and ** *P* < 0.01.

DNA yields were measured by Qubit fluorometer and NanoDrop spectrophotometer (Fig. 1). Among the extraction methods tested, Genomic-tip kit showed the highest genomic DNA yield, while TIANamp and CTAB methods resulted in the lowest genomic DNA yield (Fig. 1).

### DNA purity and integrity

Purity of extracted DNA was evaluated based on the ratios between the absorbance values for A260/280 and A260/230 using a NanoDrop spectrophotometer (Table 1). Recommended purity of DNA for a long-read sequencing platform is 1.8–2.0 for A260/280 ratio and ∼2.0 for A260/230 ratio (Hassan et al., 2015; Lucena-Aguilar et al., 2016; Usman et al., 2014). Mollusc and Genomic-tip kits were the only two methods that yielded DNA with the purity that passed the criteria for tissue sample. On the other hand, the CTAB protocol and TIANamp kit did not yield DNA samples that passed the criteria in any of the samples tested. DNA extracted using the Sbeadex livestock kit passed the criteria in A260/280, but not A260/A230 ratio (Table 1).

**Table 1:** Quality of gDNA extracted from shrimp muscle using different DNA extraction methods after purified with AMPure PB bead represented by an average ± a standard deviation value (SD).

**NanoDrop spectrophotometer**	**Extraction method**				
	CTAB	Genomic-tip 100/G kit	Mollusc DNA kit	TIANamp Marine Animals DNA kit	Sbeadex livestock kit
A260/A280	1.40 ± 0.09	1.84 ± 0.01	1.98 ± 0.04	1.63 ± 0.16	1.81 ± 0.03
A260/A230	0.40 ± 0.05	2.45 ± 0.03	2.01 ± 0.16	1.74 ± 0.22	1.85 ± 0.36

**Figure 2:**
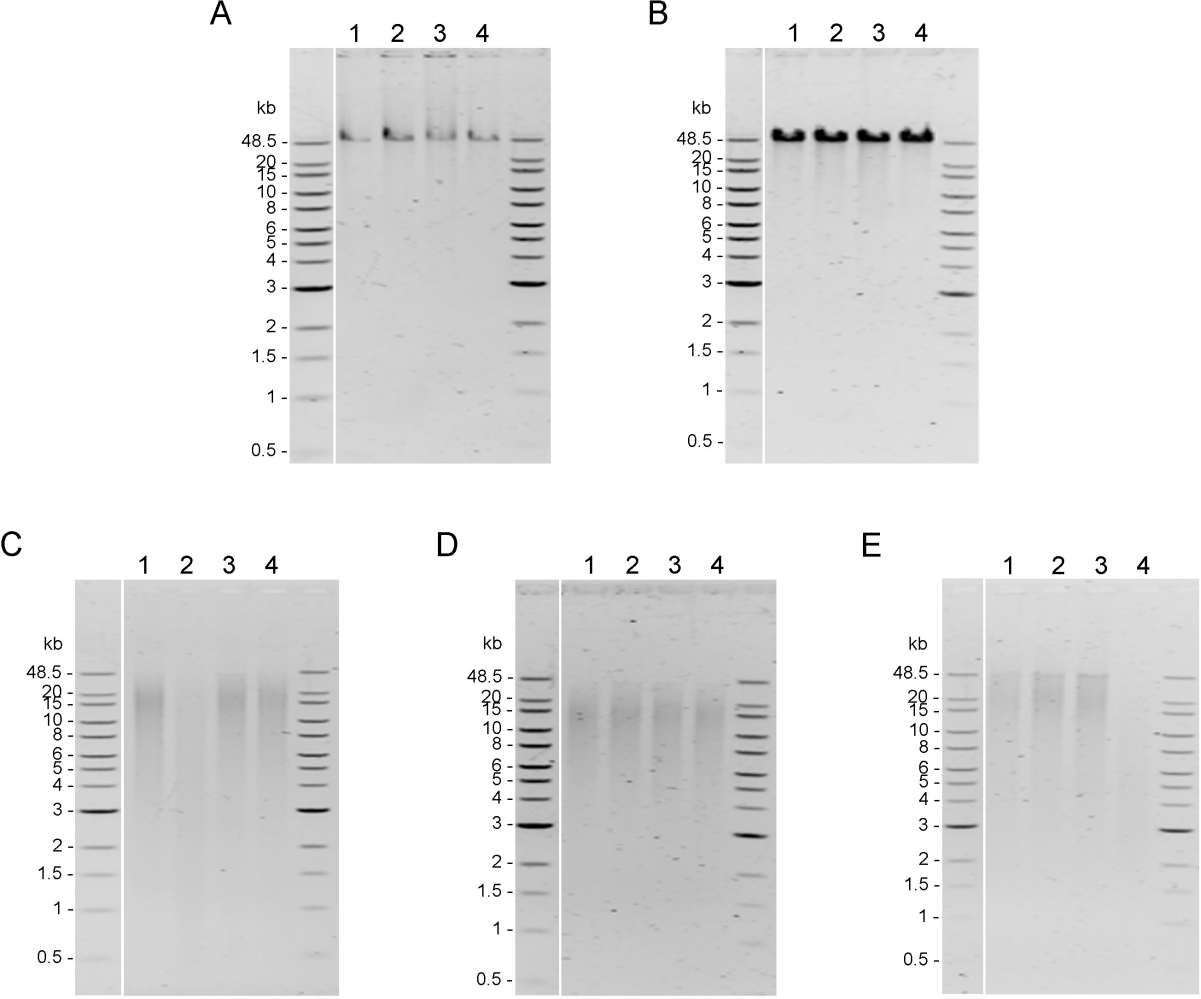
Quality assessment of genomic DNA of shrimp muscle extracted with different DNA extraction methods. After purified by AMPure PB bead, gDNA (n=4) extracted from (A) CTAB method (100 ng**** of gDNA), (B) QIAGEN Genomic-tip 100/G kit (100 ng of ****gDNA), (C) E.Z.N.A.^®^Mollusc DNA Kit (200 ng of ****gDNA), (D) TIANamp Marine Animals DNA kit (100 ng of gDNA) and (E) Sbeadex livestock kit (200 ng of ****gDNA) were loaded on 0.75% pulsed-field gel electrophoresis and run at 80 Volts for 9 h. The DNA size marker is Quick-Load 1 kb Extend DNA Ladder (New England BioLabs).

Integrity of genomic DNA samples was evaluated using pulsed-field gel electrophoresis of the 100 ng of DNA samples obtained using five DNA extraction methods after the AMPure PB bead purification step (Fig. S1). However, DNA extracted by Mollusc kit (Fig. S1C) and Sbeadex livestock kit (Fig. S1E) could not be clearly visualized; therefore, 200 ng of DNA samples from these two methods were used to improve visualization allowing better comparison of the DNA integrity in Fig. 2. DNA extracted from muscle tissue by CTAB method and Genomic-tip kit appeared to have the highest molecular weight (Figs. 2A and 2B), while the Mollusc DNA Kit, TIANamp Marine Animals DNA kit and Sbeadex livestock kit yielded DNA with smeary bands, indicating shearing of DNA (Figs. 2C, 2D and 2E).

### Sequencing quality

**Figure 3:**
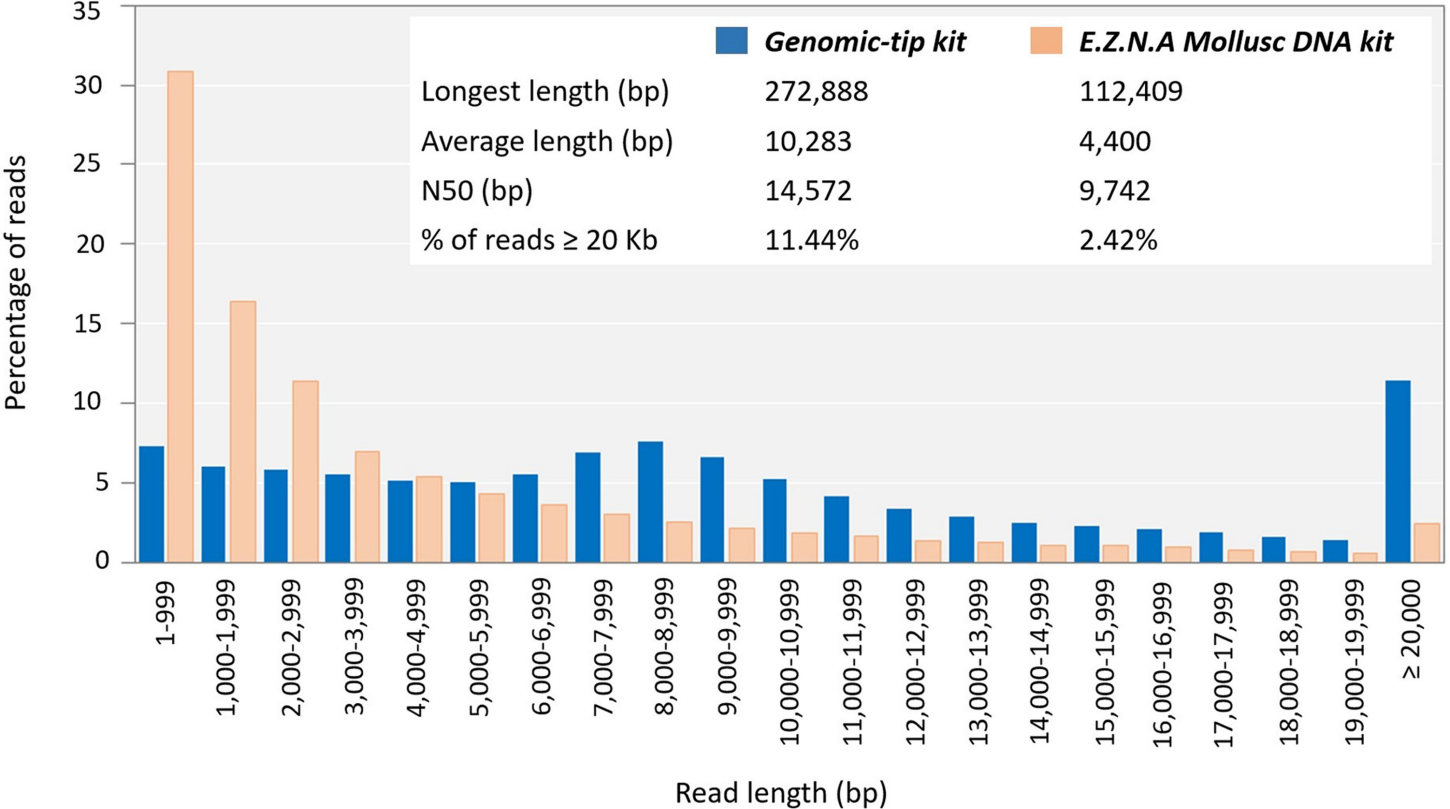
Read length distribution using DNA extracted by Mollusc DNA kit and Genomic-tip 100/G kit. Mollusc DNA kit was sequenced for two SMRT cells and Genomic-tip 100/G kit was sequenced for 37 SMRT cells.

To evaluate whether the quality of the obtained genomic DNA was sufficiently high for the long-read sequencing technology, the genomic DNA extracted by the top three extraction methods (Genomic-tip kit, CTAB method and Mollusc DNA kit) were used to prepare 20Kb libraries and subsequently sequenced using the PacBio (Fig. 3). While the genomic DNA from Genomic-tip and Mollusc DNA kits allowed successful library construction, the one from CTAB method did not. The sequencing of DNA obtained from Genomic-tip kit yielded 5.36 Gb/SMRT cell with the longest length of 272.89 Kb, an average length of 10.28 Kb and an N50 length of 14.57 Kb. The number of reads longer than 20 Kb represented 11.44% of the total reads. The sequencing of DNA obtained from Mollusc DNA kit gave 3.07 Gb/ SMRT cell with the maximum read length of 112.41 Kb, an average length of 4.40 Kb and an N50 length of 9.74 Kb. The reads longer than 20 Kb covered 2.42% of total reads.

## Discussion

Availability of genome sequences for various organisms has revolutionized biology. Recent advances in the long-read sequencing technology have promised even faster and better quality of genome sequences. With such power of the recent sequencing technology, the Earth BioGenome Project has announced to sequence, catalog and characterize the genomes of Earth’s eukaryotic biodiversity over a period of ten years (Lewin et al., 2018). While the capacity of the sequencing technology theoretically can make such a grand challenge possible, numerous organisms are intrinsically difficult to be sequenced.

Marine invertebrates are deemed to be ones of the most challenging species to be sequenced. This is possibly due to their DNA contents containing high polysaccharide content, polyphenols and other secondary metabolites which can interfere downstream DNA library preparations (Panova et al., 2016). Some of the genomes of marine organisms such as water flea (*Daphnia pulex ) (Ye et al., 2017)*, Atlantic cod (*Gadus morhua*) (Star et al., 2016), black tiger shrimp (*Penaeus monodon*) (Uengwetwanit et al., 2020; Yuan et al., 2018) and Pacific white shrimp (*Litopenaeus vannamei*) (Zhang et al., 2019) reportedly contain high density of repetitive sequences, which hinder high quality genome sequence assembly. To overcome this challenge, high quality and integrity of the starting DNA must be used to fully exploit the capacity of the long-read sequencing platform. Thus, this study embarks on comparing the efficacy of five already established protocols for genomic DNA extraction of a marine organism using the black tiger shrimp as a model.

Among the five protocols examined, CTAB method is the only one where solutions/buffers required can be prepared in-house, hence, has the advantage of being an inexpensive and simple approach for DNA extraction (Table 2). It has been successfully used to extract DNA from organisms containing a high level of polysaccharide such as plant samples (Abdel-Latif & Osman, 2017; Doyle & Doyle, 1987; Michiels et al., 2003). CTAB method also utilizes a reducing agent *β*-mercaptoethanol in lysate solution, which prevents oxidative damage of nucleic acid, resulting in HMW DNA in other organisms (Bitencourt et al., 2007; Herzer, 2001). In this study, although the DNA obtained from CTAB method remained at high molecular weight as expected, it yielded the lowest purity of DNA sample based on A260/A280 and A260/A230 ratios (Table 2). The low ratios obtained suggest ineffective removal of proteins and organic compounds. This is not surprising because the standard CTAB method does not require proteinase K treatment but uses phenol/chloroform extraction steps, which introduces organic contaminants and causes yield loss. To further improve this method for better quality of DNA, a treatment of proteinase K or other purification steps should be considered.

**Table 2:** Performance comparison of DNA extraction methods.

	CTAB	Genomic-tip	Mollusc DNA	TIANamp Marine Animals DNA	Sbeadex livestock
Time					
-Total assay time	2 h 30 min	7-8 h	17 h (overnight lysis step)	3 h	4 h
Hands-on time	1 h 30 min	1 h 30 min	1 h	45 min	2 h
Cost^a^	Inexpensive (2 USD/rnx/100 mg tissue)	Expensive (27.4 USD/rnx/100 mg tissue)	Expensive (8.2 USD/rnx/50 mg tissue)	Inexpensive (2.5 USD/rnx/30 mg tissue)	Expensive (7.2 USD/rnx/50 mg tissue)
Yield	Low yield (8.81 ng DNA/mg tissue)	Highest yield (96.38 ng DNA/mg tissue)	Low yield (13.15 ng DNA/mg tissue)	Lowest yield (8.25 ng DNA/mg tissue)	Moderate yield (56.42 ng DNA/mg tissue)
Quality					
− Integrity	HMW observed in >48.5 kb	HMW observed in >48.5 kb	Smearing of DNA observed in <20 kb	Smearing of DNA observed in <20 kb	HMW observed in 48.8 kb and smearing of DNA observed in <48.5 kb
− Purity	Poor	Good	Good	Poor	Poor

**Notes.**

a Cost indicated here was converted from the local currency to USD. It may vary in other countries depending on costs from local distributors.

The other four extraction methods are commercial kits. Genomic-tip kit employs gravity-flow, anion-exchange tips to minimize DNA shearing from centrifugation, vortexing, and pipetting steps. This gravity-flow based method gave the highest yield when compared with other methods (Table 2). This method simultaneously lyses and enzymatically digests proteins in the sample, allowing immediate denaturation of proteins such as nucleases, histones, and DNA-binding proteins (Qiagen, 2015). These proteins have been reported to affect the quality of DNA (Chacon-Cortes & Griffiths, 2014; Tan & Yiap, 2009). Shrimp muscle consists of numerous fiber proteins, majorly composed of myofibrillar and sarcoplasmic proteins (Medler & Mykles, 2015). Thus, digesting these fibrous proteins in shrimp muscle using proteinase K from the beginning may have resulted in the highest DNA yield among the extraction methods. Other fibrous samples have witnessed the same high yield benefit from performing proteinase K digestion at the beginning of the protocol as well (Vaidya et al., 2018). In addition to the high yield, the highest integrity of the DNA obtained from this kit was also evidenced by the longest read length from the sequencing result.

Mollusc kit utilizes cationic detergent, combined with polysaccharide property of CTAB and the selective DNA binding matrix. This DNA extraction kit was used for muscle of marine metazoans to remove polysaccharide contamination (Lobo et al., 2013). Using this kit with shrimp tissue gave high purity of DNA; however, the pulsed-field gel electrophoresis suggested that the DNA was sheared. The sequencing result also confirmed that the DNA from the kit was not intact as the average read length was much shorter than those from Genomic-tip kit. This kit requires the most number of centrifugation steps (7 times) when compared with other methods (CTAB method, 6 times; Genomic-tip kit, 2 times; TIANamp kit, 6 times and Sbeadex livestock kit, 1 time), which might cause the shearing of DNA (McDonald, 2002; Reigstad, Bartossek & Schleper, 2011; Sage Science, 2020).

For TIANamp kit, the mechanism for DNA extraction is very similar to that of Mollusc kit without CTAB and chloroform extraction step. The DNA obtained from this kit was one of the lowest quality and purity among the examined commercial kits. This result was indeed unexpected as the kit was successfully employed to extract genomic DNA of the Pacific white shrimp (*L. vannamei)* enabling a complete draft of genome sequence using a PacBio sequencing technology (Zhang et al., 2019). Given no details of the protocol from the paper, it might be possible that some modifications of the DNA extraction protocol were made to obtain high quality and intact DNA sufficient to give successful sequencing results.

Unlike the other commercial kits examined in this study, Sbeadex livestock kit utilizes paramagnetic microparticles whose surface binds DNA through an anion-exchange mechanism. The DNA obtained from this bead-based kit was of high purity in most samples but the integrity was compromised as observed in the pulsed-field gel electrophoresis. Due to the absence of RNaseA treatment step in this protocol, the DNA integrity might be jeopardized. Indeed, RNaseA treatment was reportedly necessary for isolation of high quality genomic DNA (Healey et al., 2014; Lienhard & Schäffer, 2019; Rana et al., 2019).

It is important to note that this study employed the established protocol for each method without further modifications. Indeed, each method can be further modified to give better extraction efficacy, resulting in better quality and yield. For example, reducing incubation time of proteinase K, which can cause DNA degradation (Athanasio et al., 2016), might result in better integrity of DNA. Optimizing animal tissues for each method can also improve digestion of the tissue to release more DNA yield (Jasrotia & Langer, 2019). The inclusion of RNaseA treatment can alleviate the issue of DNA degradation (Healey et al., 2014).

To further demonstrate the suitability for long-read sequencing of the genomic DNA obtained in this study, the DNA extracted by the top three methods (Genomic-tip kit, CTAB method, and Mollusc DNA kit) were subsequently sequenced by the PacBio technology. Genomic-tip and Mollusc DNA kits were only two methods that allowed successful library construction. This indicates that both high-molecular weight and purity of genomic DNA were equally important to successful long-read sequencing. Purity is essential to generate sequencing library, whereas the high-molecular weight of genomic DNA is critical for construction of large insert library. Despite highly intact DNA, CTAB method is not efficient at removing contaminants. Therefore, the protocol might need to be optimized for particular organism to compensate purity and quality (Arseneau, Steeves & Laflamme, 2017; Chakraborty, Saha & Ananthram, 2020).

As a matter of fact, the Mollusc kit employed in this study was a CTAB-based method that has been optimized for mollusk. However, when used with shrimp sample, it only showed improvement of purity but not integrity. Degradation of DNA might be due to long exposure of proteinase K (Athanasio et al., 2016). Mollusc kit used overnight proteinase K and RNaseA digestion, whereas CTAB method required a short incubation with RNaseA (<30 min) and Genomic-tip kit used 4 h incubation of proteinase K and RNaseA. Long incubation periods for proteinase K digestion in Mollusc kits resulted in fragmented DNA as it could be observed that 2.42% of reads were remained in read length ≥ 20 Kb in comparison to 11.44% of read length ≥ 20 Kb remaining in Genomic-tip kit. Beside longer sequencing reads (14.57 Kb of N50), Genomic-tip kit generated higher sequencing yield (5.36 Gb/ SMRT cell). Genomic-tip kit purifies DNA based on gravity-flow and anion-exchange tip, which are less prone to shearing DNA compared to vortexing, pipetting and long exposure of enzyme digestion. As demonstrated in the present study, Genomic-tip kit is a suitable method for long-read sequencing. Although Genomic-tip kit is relatively time-consuming and expensive, the high-quality and yield of sequencing reads outweigh these disadvantages (Table 2). However, for laboratories with limited resources and access, further modifications of less expensive methods such as CTAB exaction protocol might be considered.

## Conclusions

The quality of starting DNA for a long-read sequencing platform such as PacBio is a key determinant of the successful result. In this study, the efficiency of extracting high-quality HMW DNA was evaluated for five established DNA extraction methods without any modifications. It is noteworthy that further modifications of the established protocols might yield even better DNA quality and quantity. The gravity-flow based method was found to yield the highest quality genomic DNA and hence suitable for downstream whole genome sequencing application using the long-read sequencing platform. This protocol could be further used for extracting high quality genomic DNA for long-read sequencing of other marine organisms, allowing full exploitation of the sequencing platform.

##  Supplemental Information

10.7717/peerj.10340/supp-1 Supplemental Information 1 Quality assessment of genomic DNA of shrimp muscle extracted with different DNA extraction methods After purified by AMPure PB bead, 100 ng of gDNA extracted from (A) CTAB method, (B) QIAGEN Genomic-tip 100/G kit, (C) E.Z.N.A.^®^ Mollusc DNA Kit, (D) TIANamp Marine Animals DNA kit and (E) Sbeadex livestock kit were loaded on 0.75% Pulsed-field gel electrophoresis and run at 80 Volts for 9 h. The DNA size marker is Quick-Load 1 kb Extend DNA Ladder (NEW ENGLAND BioLabs).

## Additional Information and Declarations

### Data Availability

The following information was supplied regarding data availability: The data is available at NCBI: PRJNA611030 and at the Black Tiger Shrimp (*Penaeus monodon*) Genome Project (raw genomic PacBio reads which used DNA obtained from Genomic-tip kit and Mollusc DNA kit). Link “Black Tiger Shrimp (*Penaeus monodon*) Genome Project” to http://www.biotec.or.th/pmonodon/index.php/downloads.
