# Supplementary figures and images for: Optimization of high molecular weight DNA extraction methods in shrimp for a long-read sequencing platform
Source: PeerJ. 2020 Nov 13;8:e10340. doi: 10.7717/peerj.10340 (PMC7668203; doi:10.7717/peerj.10340)

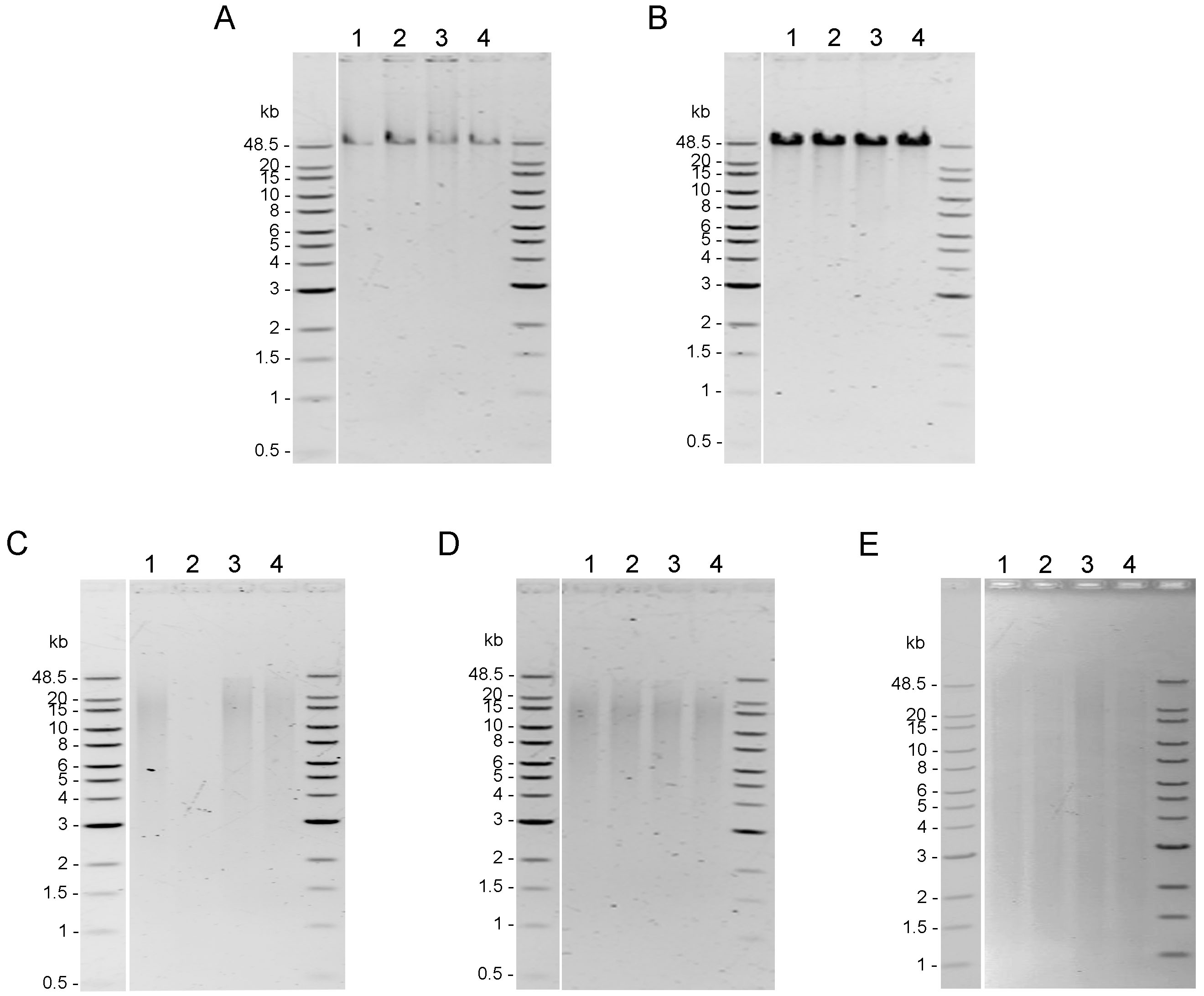

Supplement: Supplemental Information 1 — After purified by AMPure PB bead, 100 ng of gDNA extracted from (A) CTAB method, (B) QIAGEN Genomic-tip 100/G kit, (C) E.Z.N.A.® Mollusc DNA Kit, (D) TIANamp Marine Animals DNA kit and (E) Sbeadex livestock kit were loaded on 0.75% Pulsed-field gel electrophoresis and run at 80 Volts for 9 h. The DNA size marker is Quick-Load 1 kb Extend DNA Ladder (NEW ENGLAND BioLabs). [file peerj-08-10340-s001.jpg]
